# Genetic background and oncogenic driver determines the genomic evolution and transcriptomics of mammary tumor metastasis

**DOI:** 10.1038/s42003-025-08624-5

**Published:** 2025-08-14

**Authors:** Christina R. Ross, Karol Szczepanek, Jack Sanford, Gabriel Needle, Tinghu Qiu, Larry Pearce, Maxwell P. Lee, Kent W. Hunter

**Affiliations:** 1 Laboratory of Cancer Biology and Genetics, Center for Cancer Research, National Cancer Institute, NIH, Bethesda, MD USA; 2 AstraZeneca, Gaithersburg, MD USA; 3 NYU Langone Health, New York, NY USA; 4 Present Address: Johnson & Johnson Innovative Medicine, Horsham, PA USA

**Keywords:** Cancer genomics, Cancer genomics

## Abstract

Metastasis remains a major cause of cancer mortality. This study, expanding upon previous findings in the MMTV-PyMT model, investigated four independent mouse models, representing luminal (MMTV-PyMT, MMTV-Myc), HER2-amplified (MMTV-Her2), and triple-negative (C3(1)TAg) breast cancer subtypes. Consistent with previous results, limited evidence for metastasis-associated somatic point mutations was found for all models. We also found that oncogenic drivers significantly influenced the number and size of metastasis-specific copy number variations (MSCNVs), but common driver-independent MSCNVs were rare. Furthermore, analyzing a cohort with varying genetic backgrounds while maintaining a constant oncogenic driver (PyMT) revealed that genetic background profoundly impacts MSCNVs. Transcriptome analysis demonstrated that oncogenic drivers strongly shaped metastasis-specific gene expression (MSGE), with each driver exhibiting distinct expression profiles. In contrast, MSGE in the PyMT-F1 cohort was more variable across strains. Despite the diversity of MSCNV and MSGE, functional analysis revealed that both mechanisms converge on the modulation of key cellular processes, including immune responses, metabolism, and extracellular matrix interactions. These findings emphasize the complex interplay between oncogenic drivers and genetic background in shaping the genomic and transcriptional landscapes of metastatic lesions.

## Abstract (web summary)

Genetic background and oncogenic driver determines the genomic evolution and transcriptomics of mammary tumor metastasis.

## Introduction

In the United States, breast cancer is the most frequently diagnosed cancer type and accounts for the third highest number of cancer-associated deaths annually. While patients with localized disease have a 5-year survival rate of 99.6% following tumor resection, the 5-year survival rate drops to 31.9% for patients with distant metastasis^1^. This disparity stems from the inability to effectively treat secondary lesions through either surgical resection or current therapeutic strategies. Metastatic tumors reside in microenvironments and exhibit therapeutic resistances that are distinct from those of the primary tumors, warranting further research and development to create new and more specific therapeutic strategies.

Unfortunately, despite many recent advances, the etiology and mechanisms driving metastasis remain unclear. The conventional model of metastatic progression was first proposed by Peter Nowell in 1976, who hypothesized that tumors arise from a single mutated cell and continue to acquire additional mutations that persist if they enable survival and growth^2^. In this model, clones continue to evolve within the primary tumor (PT), ultimately developing into subclones with metastatic ability. Recent increased access to DNA-sequencing technologies has enabled several clinical studies to characterize the genomic landscape of metastatic tumors^3–5^. However, although much of the data obtained to date is consistent with this hypothesis, no metastasis-driver mutations or acquired genomic aberrations have been identified in any cancer type. Indeed, current data suggest the acquisition of transcriptomic and epigenetic switching mechanisms, rather than mutations, drive dissemination and secondary tumor growth^6–9^.

To better understand the etiology of breast cancer metastasis, well-credentialed, physiologically relevant, and accessible experimental systems are required. Clinical data, while extraordinarily valuable, is difficult and expensive to obtain in large numbers, is experimentally limited, and is usually confounded by treatment, which may significantly influence data interpretation. Mouse models of metastatic cancer, therefore, provide a complementary experimental vehicle to assess the etiology of metastasis in a treatment-naïve, highly controlled setting. Indeed, several genetically engineered mouse models (GEMMs) have been developed to mimic the molecular subtypes of metastatic breast cancer found in patients. Our laboratory and many others have published extensively using the mammary-specific polyomavirus middle T antigen overexpression mouse model (MMTV-PyMT) to model the luminal B subtype of human breast cancers^6,10–12^. This model was developed in 1992 and faithfully recapitulates human breast cancer progression and metastasis to the lung^13,14^.

Previously, we performed genomic and transcriptomic analysis of the MMTV-PyMT model. By performing whole exome-sequencing (exome-seq) on genomic DNA (gDNA) from matched PT and lung metastatic (lung met) tissues, we showed that very few single-nucleotide variants (SNVs) occur in metastases at a high frequency that are not also found in the primary tumor and concluded that SNVs are an unlikely driver of metastasis^6^. In addition, we observed no obvious metastasis-associated translocations, but some metastasis-associated copy number variation (CNV). In this study, we expand upon this work to include both the MMTV-Myc and the C3(1)TAg models, as well as provide greater depth to our prior analysis of the MMTV-Her2 and PyMT models. In addition, we have further investigated how an individual’s personal ancestry might influence metastasis-associated genomic evolution in the MMTV-PyMT model by analyzing F1 animals generated from crosses between phylogenetically related mouse strains that exhibit significant differences in metastatic efficiency. Consistent with previous analyses, we confirm that SNVs with a metastasis driver function are not present in most of the animals analyzed, regardless of oncogenic driver or genetic background. However, we have identified genetic background as a strong determinant of PT stability, copy number region size, and gene-set enrichment within regions of gDNA gain or loss. Further, we show that gene expression at end-stage is not strongly correlated with CNV in our models. Finally, our work reveals no common gene or gene-set found within CNV, but rather cellular processes enriched in metastasis-specific gene expression and alternative splicing.

## Results

### Single nucleotide variants are unlikely drivers of metastasis

**Fig. 1:**
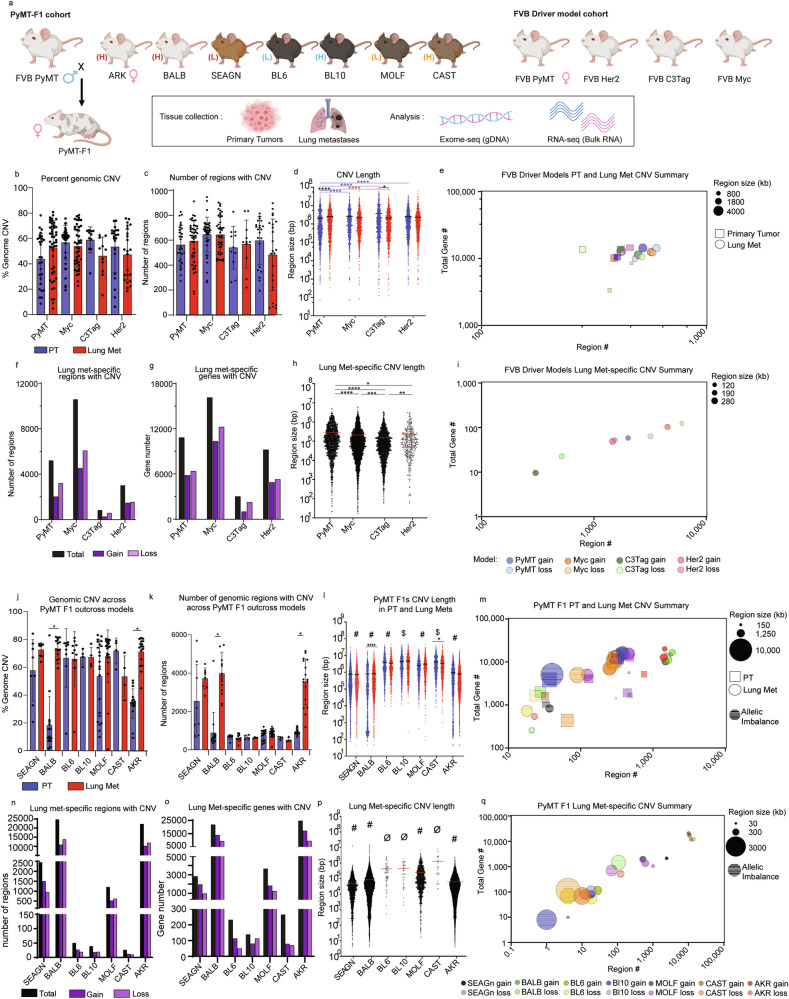
Model and genetic background shape MSCNV. **a** Schematic overview of mouse models, tissues collected, and analyses performed in this study. Histograms showing **b** the average percentage of the genome and **c** the average number of regions with CNV with standard deviation (SD), PT (blue), and lung mets (red). **d** Dot plot of individual CNV region lengths from aggregate data (the average indicated by black line). **e** Multivariate bubble plot showing average CNV number and aggregate total number of genes with CNV for PT (squares) and lung met (circles) from each model, with bubble size corresponding to CNV region size. Total number (black) of regions (**f**) and genes (**g**) with significant CNV gain (dark purple) and loss (light purple) in MSCNV. **h** Dot plot showing individual CNV region lengths from MSCNV (the average indicated by red line). **i** Multivariate bubble plot showing average CNV number and aggregate total number of genes with CNV for MSCNV (circles) from each model, with bubble size corresponding to CNV region size. **j** Histogram showing the average % of the genome and **k** average number of regions with CNV with SD, PT (blue), and lung mets (red). **l** Dot plot showing individual CNV region lengths from aggregate data with all animals per model combined (the average indicated by a line). **m** Multivariate bubble plot showing average CNV number and aggregate total number of genes with CNV for PT (squares) and lung met (circles) from each model, with bubble size corresponding to CNV region size. **n** Total number (black) of regions and **o** genes with significant CNV gain (dark purple) and loss (light purple) in Lung mets compared to PT. **p** Dot plot showing individual CNV region lengths from MSCNV (the average indicated by red line). **q** Multivariate bubble plot showing average CNV number and aggregate total number of genes with CNV for MSCNV (circles) from each model, with bubble size corresponding to CNV region size. Significance determined by Kruskal–Wallis test (**d**, **h**, **l**, **p**) or unpaired *t* test (**b**, **c**, **j**, **k**). *(*p* < 0.05), **(*p* < 0.01), ***(*p* < 0.001) ****(*p* < 0.0001), $ = significantly different from 6 groups, # = significantly different from 5 groups, ø = significantly different from 4 groups. Fig. 1a was generated using BioRender.

To evaluate the relative contributions of oncogenic driver and genetic background to the genomic landscape of primary tumors and lung metastases, we developed two mouse model cohorts (Fig. 1a). The first cohort, the “oncogenic driver” cohort, utilized four distinct mouse models of metastatic breast cancer, each with a unique oncogenic driver but identical FVB genetic background. The mouse mammary tumor virus (MMTV)-neu (Her2) line was used to model Her2-amplified (Her2+) breast cancer, a subtype representing approximately 17% of breast cancer patients^15,16^. The MMTV-Myc (Myc) line was used to model metastatic Myc-driven breast cancer, which is observed in approximately 30–50% of high-grade tumors^17^. The C3(1)/TAg-REAR (C3Tag) line was used to model a metastatic basal-like subtype, which is seen in 8% of breast cancer patients^18,19^. Finally, the N-Tg(MMTV-PyVT)634Mul/J (PyMT) line^20^, which forms tumors that are highly metastatic, was used to model activated tyrosine kinase-driven cancers, which represent 49% of Luminal B, 32% of Luminal A, 42% of Her+, and 7% of Basal-like tumors, or 40% of all breast cancers^21^. Thus, the “oncogenic driver” cohort was comprised of four groups, PyMT, Her2, C3Tag, and Myc, with the FVB genetic background held constant. For the second cohort, or “PyMT-F1” cohort, FVB-PyMT males were bred to females from seven different genetic backgrounds and tumor-bearing PyMT-F1 offspring were evaluated. The genetic backgrounds chosen constitute two pairs (MOLF and CAST, BL6, and BL10) and one group of three (AKR, BALB, SEAGN) closely related strains with inverse metastatic efficiency (high (H) or low (L)) (Fig. 1a and Supplementary data 1)^12^, permitting us to evaluate the contribution of genetic background on the genomic landscape with oncogenic driver held constant. We isolated genomic DNA (gDNA) from matched pairs of primary tumors (PT) and lung metastases (Lung mets) from both cohorts (Supplementary data 1) and performed exome-sequencing (exome-seq) (Fig. 1a).

We have previously shown that high allele frequency, metastasis-specific SNVs were not found at consequential rates in a smaller mouse cohort^6^. Using the exome-seq data from this larger cohort described above, we expanded the number of individuals used for SNV analysis. Consistent with our previous data, high frequency, metastasis-specific SNVs were not observed, suggesting that common metastasis driver mutations, analogous to common tumor driver mutations, are not major drivers of tumor progression (Supplementary fig. 1). However, as previously demonstrated^6^, low frequency metastasis-specific SNVs were observed, suggesting that either there are many low frequency metastasis driver SNVs or subsets of patients in which metastasis-specific SNVs contribute to tumor progression. However, due to the low frequency of the potential metastasis driver SNVs, subsequent analysis focused on copy number variation (CNV) induced by regions of genomic gain or loss to determine whether genome rearrangement functioned as more frequent metastasis driving events. Herein, through use of Nexus Copy Number Software 10.0, we have evaluated CNV differences across sites (PT or Lung mets), both within and between cohorts.

### Oncogenic driver has little influence over CNV event number

We first evaluated the oncogenic driver cohort, visualizing PT and Lung met data separately for each FVB model, and calculated the average percent of the genome with CNV (Supplementary data 2). We observed no significant difference in the percentage of the genome with DNA gain or loss between PT and Lung mets, and no difference between oncogenic driver models (Fig. 1b). The average percentage of the genome with CNV ranged between 43–58% in PTs and 46–53% in Lung mets (Fig. 1b and Supplementary fig. 2). Similarly, the average number of regions with CNV did not differ significantly between tissue type or model (Fig. 1c and Supplementary data 2). To determine whether the high degree of CNV observed was the result of whole genome duplication, the samples were reanalyzed using the ABSOLUTE algorithm^22^. Unlike human tumors^23^, no evidence whole genome duplication was observed in the mouse samples.

Despite having a similar degree of CNV overall, we observed key differences in the size of DNA regions with amplification or deletion. While no significant differences were noted in the CNV length for the Myc and Her2 models, CNV regions in the PTs were shorter in PyMT and longer in C3Tag compared to their matched Lung mets (Fig. 1d and Supplementary data 3). When analyzing regions of gain or loss separately, we found that regions of DNA gain were significantly larger (3572–4057 kb) than those of loss (806–1340 kb) in PTs from three models (Her2, Myc, and PyMT), and in Lung mets from all models (2497–3483 kb gains, 1370–2330 kb losses) (Supplementary fig. 3a, b). However, despite these slight but consistent differences in CNV gain and loss length, the four FVB oncogenic driver models maintained a similar number of regions and genes impacted by CNV (Fig. 1e).

### Oncogenic driver determines metastasis-specific CNV event number and size

We next focused on metastasis-specific CNV (MSCNV) for each model, or regions altered in Lung mets but not PTs. Interestingly, oncogenic driver played a significant role in the number of MSCNV regions and the number of genes within them (Supplementary data 4). The Myc driven model had the highest number of MSCNV regions (10,576) impacting the highest number of genes (16,134), followed by PyMT (5198 regions, 10,823 genes), Her2 (3003 regions, 9213 genes), and C3Tag (818 regions, 3007 genes) (Fig. 1f, g and Supplementary data 4, 5). Regions of MSCNV were even shorter in length than the average Lung met CNV by around 10-fold, 200 kb compared to 2000 kb, respectively (Fig. 1h and Supplementary fig. 3c). Overall, there were small but significant differences in the average length of MSCNV between models (Fig. 1h). When evaluating CNV gain and loss separately, we again observed significant differences in the length of MSCNV regions in a model-dependent manner (Supplementary fig. 3c and Supplementary data 4). Myc and C3Tag MSCNV regions of gain were significantly longer than regions of loss, while the opposite was observed for PyMT, and no difference was observed for Her2. This data reveals an oncogenic driver-dependent influence on MSCNV number and size, thus impacting the number of genes with Lung met-specific CNV (Fig. 1i).

### Genetic background alters CNV event number and size

We next performed similar analyses on samples from our PyMT-F1 cohort to evaluate the contribution of genetic background to CNV when the oncogenic driver (PyMT) was held constant (Fig. 1a and Supplementary data 1). While the percentage of the genome impacted by CNV was similar in Lung met tissue across strains (53–73%), significantly less was observed in PTs from BALB and AKR F1 mice, at only 18.24% and 33.89%, respectively (Fig. 1j, Supplementary fig. 4a and Supplementary data 2). More specifically, BALB and AKR had fewer regions of CNV within PT gDNA compared to matched Lung met tissue (Fig. 1k). Interestingly, when comparing the number of regions of Lung met CNV between all F1s we noted a divergence where gDNA from BL6, BL10, MOLF, and CAST tissues had between 600 and 900 regions, but gDNA from BALB, SEAGN and AKR had 3000–4000 regions (Fig. 1k). Due to the heterozygous genetic background of the F1 PyMT mice, we were also able to assess regions of allelic imbalance. While gDNA from most strains had a similar number of regions with allelic imbalance in PT and Lung mets (18–40), CAST and MOLF, closely related wild-derived strains, had a much higher number (65–128) (Supplementary figs. 3d, 5 and Supplementary data 2).

When measuring CNV region size in tissue from PyMT F1s we observed an inverse relationship with region number. BL6, BL10, MOLF, and CAST F1 mice had larger regions of CNV than SEAGN, BALB, and AKR (Fig. 1l and Supplementary data 3). We theorized that this inverse relationship was likely responsible for the relatively constant percentage of the genome impacted by CNV, excluding BALB and AKR PTs. In addition to differences between strains, we also observed small but significant differences in the length of CNVs in Lung mets compared to PTs in CAST and BALB F1s (Fig. 1l). Analysis of region size was further broken down to genomic gains, losses, and allelic imbalance (Supplementary fig. 3e, f). Regions of genomic loss trended larger than those of gain in both PT and Lung mets, regardless of background (Supplementary fig. 32f). Further, in some F1 strains, the regions of allelic imbalance in PT and Lung met gDNA were significantly larger than the average CNV size. In strains with larger allelic imbalance, the size was roughly double the length of average CNV (Supplementary fig. 3e, f and Supplementary data 3). Taken together, PyMT F1 models have unique CNV fingerprints shaped by CNV region number and region size, therefore altering the number of genes impacted by CNV (Fig. 1m).

### Genetic background alters metastasis-specific CNV event number and size

We next focused on MSCNV among the PyMT-F1 cohort. First, the closely related BL6 and BL10 strain pair had the fewest number of MSCNV, with only 50 and 39, respectively (Fig. 1n and Supplementary data 4). Closely related BALB, SEAGN, and AKR had the highest number of MSCNV, ranging from 2448 to 24,650. Though both MOLF and CAST are wild-derived strains, they exhibited vastly different numbers of MSCNV regions, at 1201 and 26, respectively. Interestingly, the number of regions of allelic imbalance within MSCNV did not appear to correlate with strain-relatedness and ranged from a single region observed in BL10 F1s to 121 regions in AKR F1s (Supplementary fig. 3g).

As we observed with the oncogenic driver models (Fig. 1g), the number of genes impacted by MSCNV correlated closely with the number of regions, rather than the size of CNV region. BL6, BL10, and CAST had the smallest number of genes impacted by CNV, ranging from 139 to 264 (Fig. 1o and Supplementary data 5). BALB, SEAGN, and AKR had the highest number of genes contained within MSCNV, ranging from 2820 to 24,615 (Fig. 1n). Further, the number of genes altered by CNV appeared to be evenly distributed between regions of gain and loss. Finally, the number of genes found within regions of metastasis-specific allelic imbalance also correlated with the number of regions rather than region size (Supplementary fig. 3g-i).

MSCNV region size again followed an inverse relationship with region number. SEAGN, BALB, and AKR had the smallest regions of MSCNV, ranging from 49 to 83 kb, whereas CAST had the largest average size of 1250 kb (Fig. 1p and Supplementary data 4). Comparison of MSCNV length between F1 strains revealed that each strain had a statistically different MSCNV length from all other F1s, and only BL6 and BL10 were not statistically different from one another. When region size for MSCNV was further broken down to gDNA gain, loss, and allelic imbalance, we noted once again that regions of allelic imbalance were larger than gain or loss for six of the seven F1 strains, a difference that was statistically significant in BALB and AKR (Supplementary fig. 3i). Metastasis-specific regions of gain and loss varied greatly in size across F1 models, but there was little difference in size when comparing regions of loss and gain within the same F1 strain (Supplementary fig. 3i). Together, this data reveals that metastasis-specific CNV follows a linear trend between CNV region number and region length, as well as between region number and the number of genes with CNV, which is modulated in a strain-dependent manner (Fig. 1q).

### Genetic background shapes gene set enrichment in CNV

**Fig. 2:**
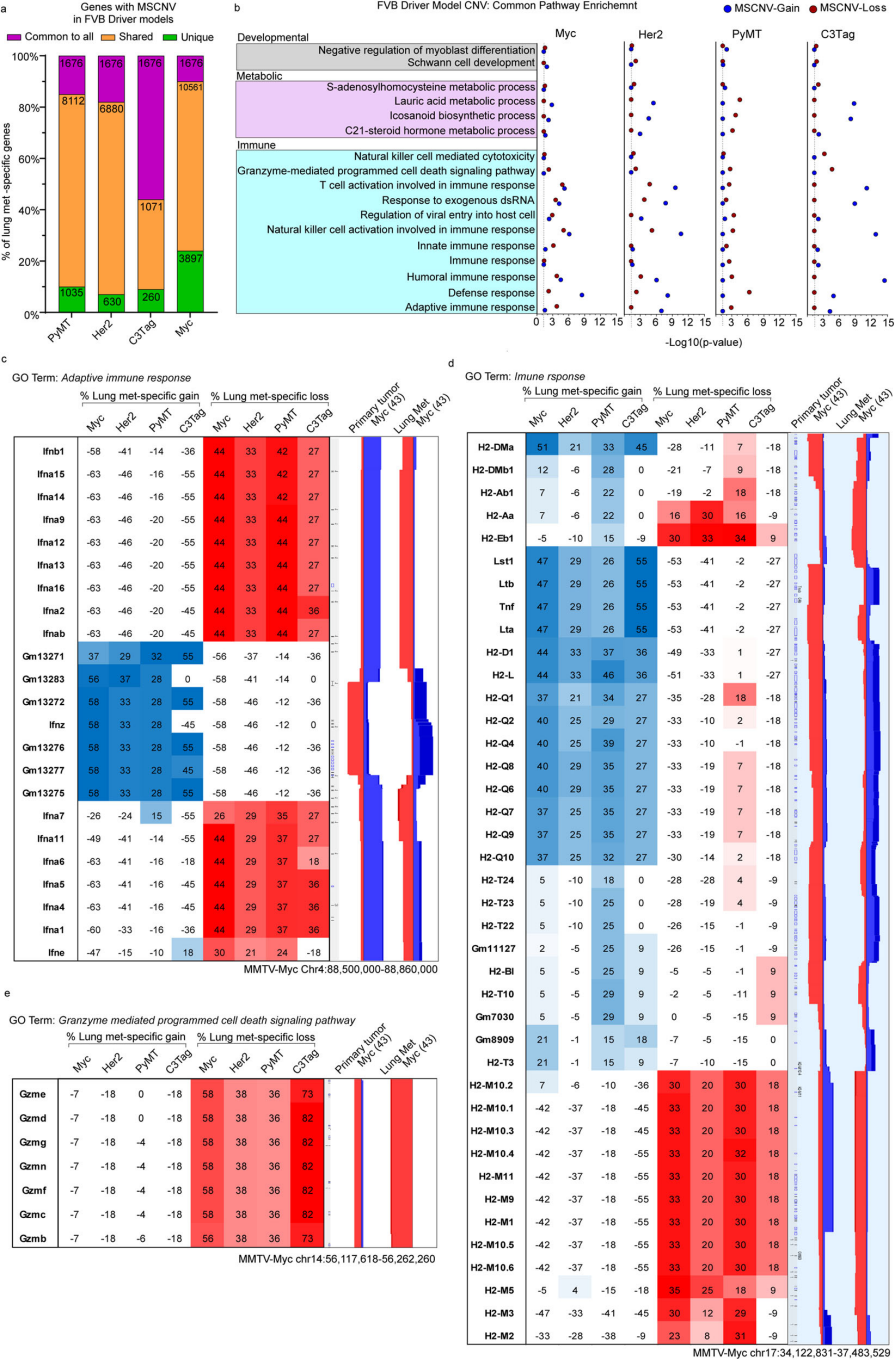
FVB driver models have metastasis-specific regions and gene families with CNV. **a** Histogram showing the percent overlap in MSCNV gene lists from each model. Numbers within bars indicate gene number common to all (purple), shared among some (yellow), or unique to each model (green). **b** DAVID analysis clustering of 1676 MSCNV genes common to all models with enrichment in CNV gain (blue) or loss (red) indicated by −Log10(*p* value) (dotted line indicates cutoff for significance (≥1.3). Heatmap indicating % lung met-specific gain in blue (% PT gain − % LM gain) and lung met-specific loss in red (% PT loss − % LM loss) for genes within a single genomic region from the **c** Adaptive immune response, **d** immune response, and **e** Granzyme mediated programmed cell death signaling pathway. CNV signal traces from PT and lung mets aligned using Nexus 10 genomic view and the Myc model data set.

We next determined if specific genes or pathways were commonly enriched in MSCNV. For the FVB oncogenic driver models, 1676 genes were commonly found within regions of MSCNV across all four models, which accounted for 10-56% of the overall number of genes within MSCNV in each model (Fig. 2a and Supplementary fig. 6a). Gene ontology (GO) analysis of this common gene set revealed enrichment of cell adhesion, granzyme-mediated cytolysis, and T-cell viral response pathways (Supplementary fig. 6b and Supplementary data 6). Comparison of GO analysis performed with the MSCNV gene sets from each FVB model individually revealed 25 pathways commonly impacted by MSCNV: 11 immune-related, four metabolic, two developmental, and eight uncategorized (Fig. 2b, Supplementary fig. 6c and Supplementary data 7). Notably, one of the uncategorized pathways was *Cellular response to nicotine*, which we previously identified in a prior large transcriptomic study and showed that exposure to nicotine significantly increased mammary cancer metastasis in a mouse allograft model^11^.

Interestingly, several of the immune-related pathways contained CNV-altered gene families clustered within a single locus. Furthermore, pathways enriched by CNV had both significant metastasis-specific gain and loss (Fig. 2b). A 400 kb region of chromosome 4 was altered in six of the 11 immune-related pathways common to the oncogenic driver cohort MSCNV. Within this region, metastatic samples had genomic gain flanked on either side by genomic loss, but the inverse was observed in PTs (Fig. 2c). This locus contains a cluster of 23 interferon genes similar to a region housing several ortholog interferon genes on human chromosome 9 p21.3. Additionally, we identified a 3000 kb region on chromosome 17 with both metastasis-specific gain and loss. Within this locus are the 32 H2 genes that make up the major histocompatibility complex (MHC). Specifically, we observed a common amplification of H2-Q genes and deletion of H2-M genes in metastases and the inverse in PTs (Fig. 2d). A third region of interest was observed on chromosome 14 that stretched approximately 150 kb and included seven granzyme (Gzm) genes that can be found within two of the common immune pathways. We observed significant Lung met-specific deletion of this locus across all four FVB driver models (Fig. 2e). While several more loci were identified as major contributors to each enriched gene ontology, we noted that PyMT and Myc often aligned, but C3Tag and Her2 samples were more variable (Supplementary data 8).

**Fig. 3:**
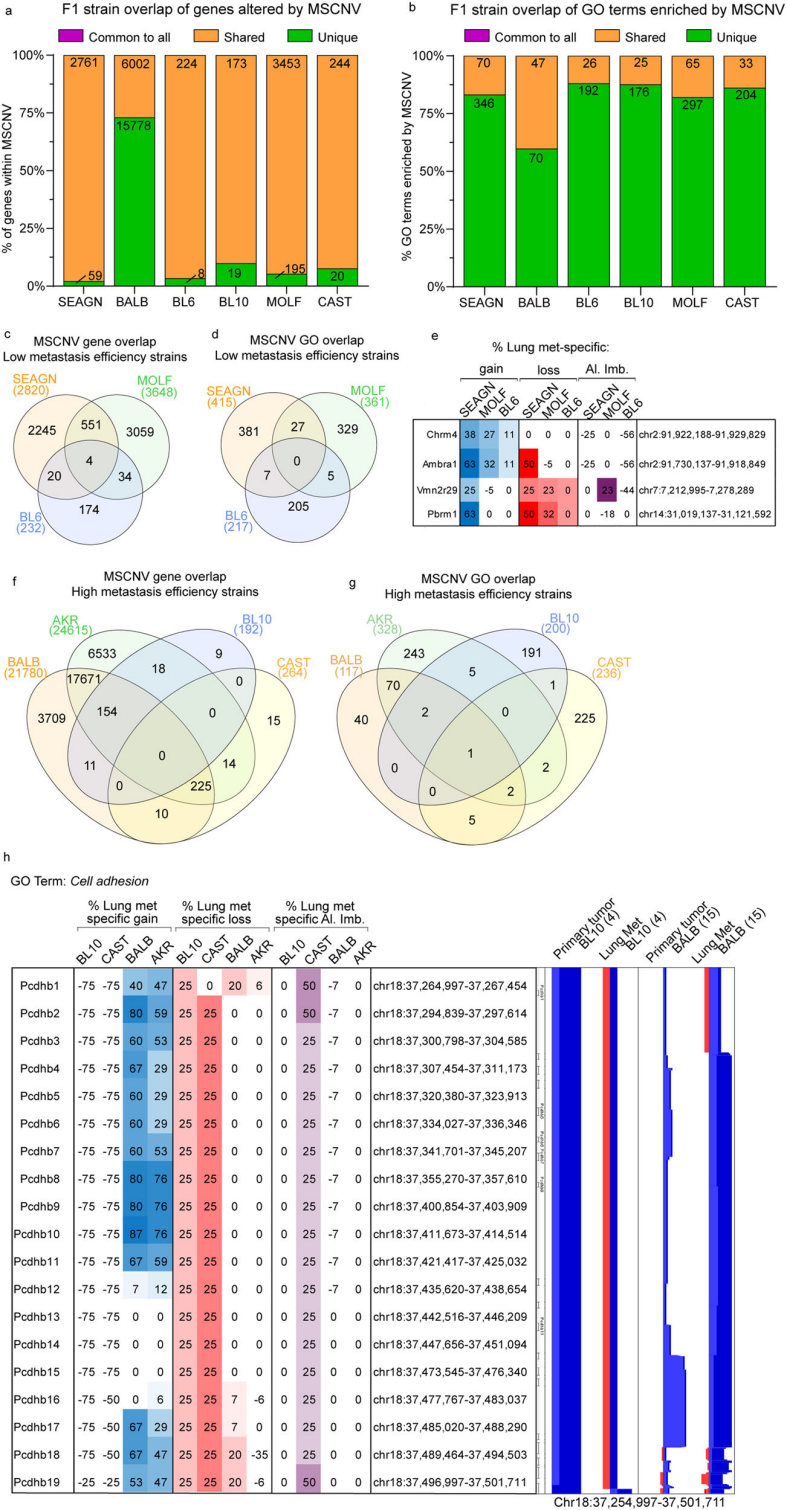
PyMT F1 models have no common gene sets or ontologies enriched by MSCNV. Histograms of the percent overlap in gene lists (**a**) and GO terms (**b**) enriched by MSCNV in each model. Numbers within bars indicate gene number common to all (purple), shared among some (yellow), or unique to each model (green). Venn diagram analysis of gene sets (**c**) and GO terms (**d**) enriched by MSCNV in low metastatic efficiency strains. **e** Heatmap indicating % lung met-specific gain in blue (% PT gain − % LM gain) and lung met-specific loss in red (% PT loss − % LM loss) for genes with MSCNV in low metastatic efficiency strains. Venn diagram analysis of gene sets (**f**) and GO terms (**g**) enriched in MSCNV from high metastatic efficiency strains. **h** Heatmap indicating % lung met-specific gain in blue (% PT gain − % LM gain), lung met-specific loss in red (% PT loss − % LM loss), and lung met-specific allelic imbalance (Al. Imb.) in purple (% PT Al. Imb. − % LM Al. Imb.) for genes with MSCNV in high metastatic efficiency strains from the Cell adhesion pathway within a single locus. CNV signal traces from PT and Lung mets aligned using Nexus 10 genomic view of the BL10 and BALB model data sets.

We performed a similar analysis for the PyMT-F1 cohort. The 25 common pathways identified from the FVB oncogenic driver model analysis were compared to GO terms enriched in MSCNV from each F1 model individually using several two-group Venn diagram analyses. AKR had the highest overlap of 17 pathways, followed by BALB (6), SEAGN (2), and MOLF (1), while BL6, BL10, and CAST had no overlap (data not shown), suggesting that genes within regions of MSCNV are enriched in a genetic background-dependent manner, as F1s with the highest GO overlap were also the strains most closely related to FVB. Additionally, comparative analysis of gene lists from regions of MSCNV revealed that, except for in BALB F1s, most genes were subject to CNV in more than one strain but no genes were commonly altered in all strains (Fig. 3a, Supplementary fig. 6d and Supplementary data 5). Similarly, comparison of gene ontologies enriched by CNV were largely unique to each PyMT-F1 strain and there were no GO terms enriched by MSCNV across all strains (Fig. 3b, Supplementary fig. 6e and Supplementary data 9).

We previously reported differing metastatic efficiency of the mouse strains included in this study (Fig. 1a and Supplementary data 1)^12^. Therefore, we next asked if F1-PyMT metastases from strains with either high or low metastatic efficiency might have similar genomic evolution and common MSCNV. To test this, genes within CNV gain, loss, and allelic imbalance, as well as enriched GO terms were compared among strains with low (SEAGN, BL6, and MOLF) and high (BALB, AKR, BL10, and CAST) metastatic efficiency. For low metastatic efficiency strains, we identified four genes commonly altered by MSCNV, but no commonly enriched GO terms (Fig. 3c, d). The *Chrm4* and *Amrba1* genes are found within a region of metastasis-specific genomic gain on mouse chromosome 2 (human chr11 p11.2), whereas *Vmn2r29* and *Pbrm1* are found on mouse chromosome 7 and 14, respectively, and have MSCNV loss (Fig. 3e). In contrast, high metastatic strains revealed no common genes within regions of MSCNV, but all strains had enrichment of the *Cell adhesion* GO term (Fig. 3fg). Heatmap analysis showed high variability between strains for genomic gain or loss of factors within the *Cell adhesion* ontology gene list, except for a locus on mouse chr18 encompassing a stretch of 19 protocadherin genes (Fig. 3h and Supplementary data 4). BALB and AKR showed amplification of this region in metastatic tissue compared to PTs, whereas BL10 and CAST showed amplification within the PT DNA not seen in the metastases, therefore resulting in a comparative metastasis-specific loss in this region (Fig. 3h).

As well as genes within regions of loss and gain, we assessed imbalance between the FVB and non-FVB allele induced by CNV and enriched in Lung mets. Allelic imbalance of a common gene or gene set was not observed across this cohort (Supplementary fig. 7a). Additionally, we did not observe allelic imbalance common to strains with either high or low metastatic efficiency (Supplementary fig. 7b, c). However, when we observed allelic imbalance within each closely related strain pair, common regions of imbalance were identified. In BALB and SEAGN PyMT F1 mice we observed common allelic imbalance in two regions, Chr1qH3 and Chr11qB4 (Supplementary fig. 7d). Interestingly, four genes found within a region of amplification on Chr1qH3 had an allelic imbalance in BALB Lung mets that was not observed in the PTs, but SEAGN showed the inverse with allelic imbalance in the PT not observed in Lung mets. The second region encompassed three genes with allelic imbalance in both BALB and SEAGN PTs that was not enriched in Lung mets (Supplementary fig. 7d). These seven genes are similarly arranged within the human genome at loci Chr1 q23.3 and Chr17 p13.2, respectively. MOLF and CAST PyMT F1s also had two common regions of metastasis-specific allelic imbalance. A region of PT and Lung met genomic gain on Chr7q resulted in allelic imbalance in Lung mets from both strains (Supplementary fig. 7e). This locus houses 11 genes and corresponds to the human Chr10q26 which contains the same set of ortholog genes. A second region within mouse Chr18q was also a site of genomic gain and common Lung met-specific allelic imbalance, encompassing 10 genes that are also found together within human Chr5q31.3 (Supplementary fig. 7e). The third strain pair, BL6 and BL10, both had metastasis-specific allelic imbalance, but no common regions could be identified (Supplementary fig. 7f). Taken together, this data suggests that genetic background is a stronger determinant than oncogenic driver in shaping the enrichment of CNV-altered genes in the metastatic genome.

### Metastatic gene expression is not shaped by CNV

**Fig. 4:**
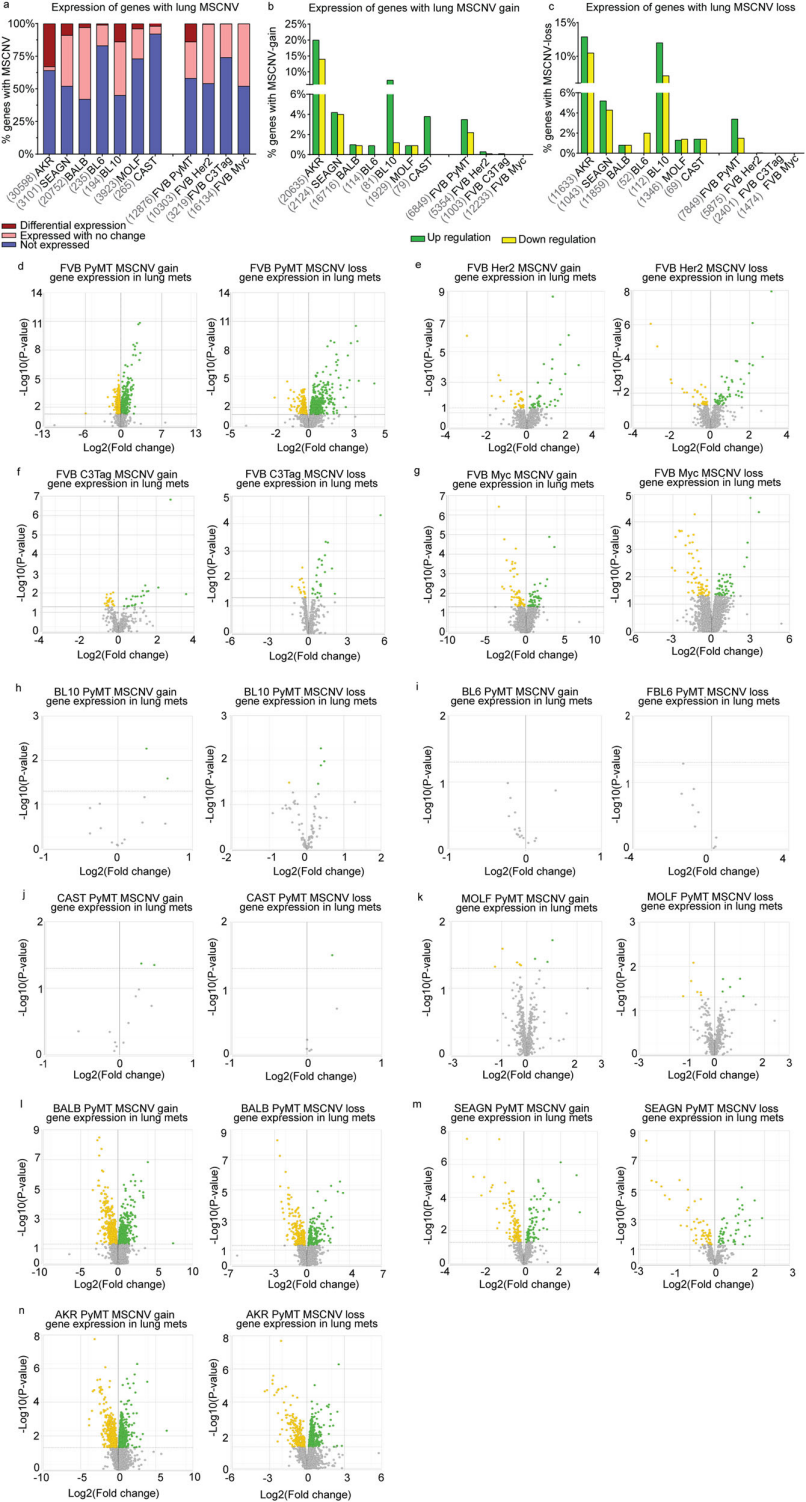
MSCNV does not significantly impact metastasis-specific gene expression. **a** Histogram of the percentage of genes with MSCNV that are not expressed (blue), expressed in lung mets with no change from PT (pink), and differentially expressed in lung mets compared to PTs (red). Numbers listed before each model name represent the MSCNV gene set size. Histograms showing the percentage of genes with MSCNV-gain (**b**) or MSCNV-loss (**c**) that had significant up regulation (green) or down regulation (yellow) in lung mets compared to PTs. Paired volcano plots for the MSGE of genes within regions of MSCNV gain or loss for each FVB driver model (**d**–**g**) and PyMT-F1 model (**h**–**n**).

We next asked if genes in MSCNV regions were differentially expressed in the metastatic lesions by performing bulk RNA-sequencing (RNA-seq) of RNA isolated from matched PT and Lung met tissue collected from the oncogenic driver and PyMT-F1 cohorts. We found significant variation in the number of genes within regions of CNV that were expressed in metastatic tumors (Fig. 4a and Supplementary data 10). CAST had the lowest expression with only 8% of MSCNV genes found in the RNA-seq signal, but BALB had the highest with 58%. We next performed differential gene expression analysis between Lung mets and PTs for each model and asked if genes with MSCNV had altered expression compared to PTs (Supplementary data 11). Again, we observed high variability within the data, with the FVB-MYC model showing the lowest number of MSCNV genes with altered expression (0.01%), and PyMT F1-AKR showing the highest (33%). When we examined the genes within MSCNV that also had significant differential gene expression compared to the PT, we noted that the expression change did not correspond to the CNV type (gain or loss) (Fig. 4b, c). Taken together this shows a poor association between CNV type and direction of any changes in gene expression at the macro metastasis stage (Fig. 4d–n).

### Oncogenic driver and genetic background shape metastatic gene expression

**Fig. 5:**
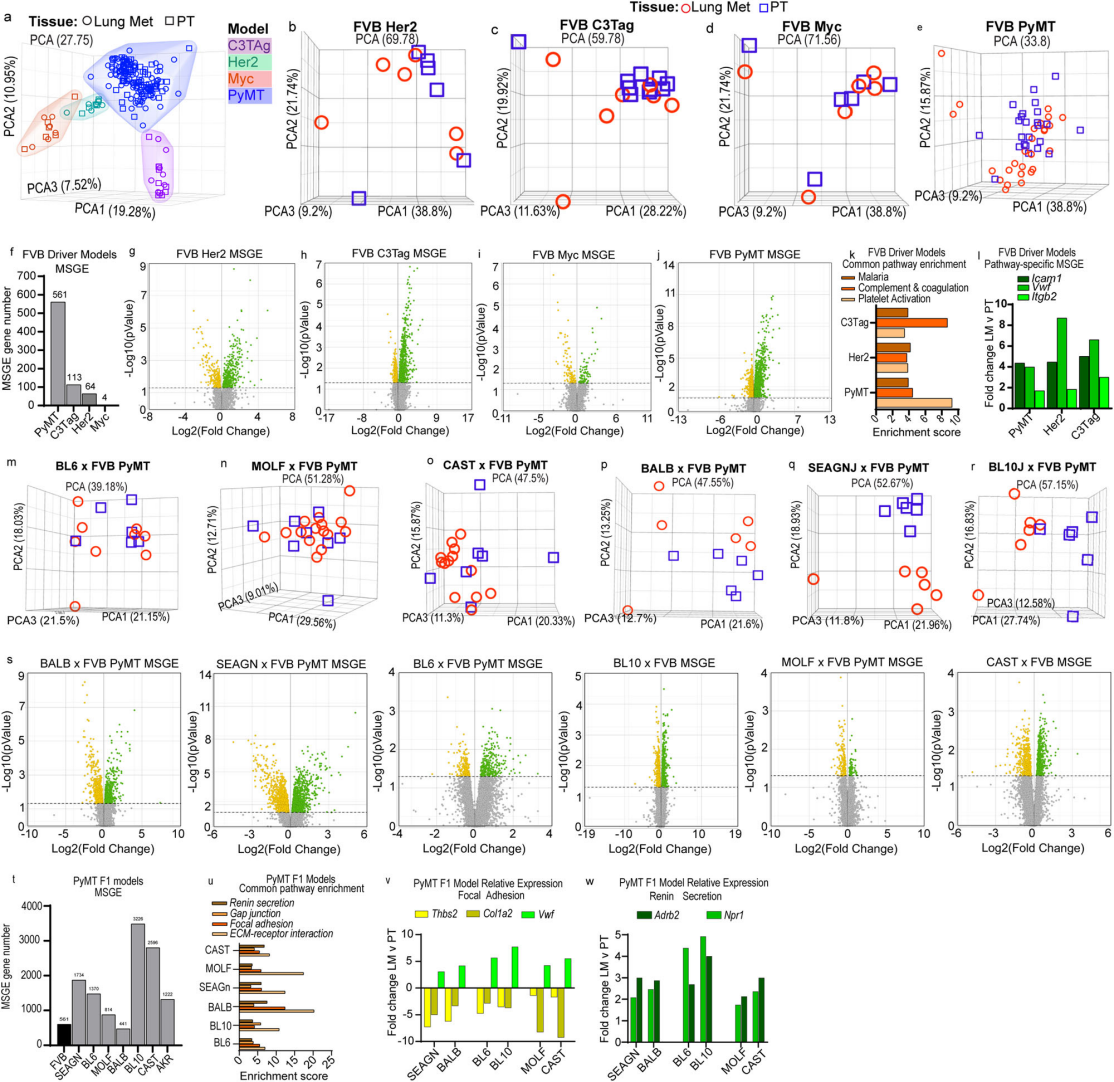
Metastatic gene expression is shaped by oncogenic driver and genetic background. **a** Unsupervised PCA of RNA-seq data from PT and Lung met tissues across all FVB driver and PyMT-F1 mouse models. Unsupervised PCA of individual PT and lung met samples from FVB Her2 (**b**), FVB C3Tag (**c**), FVB Myc (**d**), and FVB PyMT (**e**). **f** Histogram of the number of alternatively expressed genes in lung mets vs. PT for each FVB driver model (FDR < 0.05). Volcano plots with significantly up regulated genes in green and down regulated genes in yellow for MSGE in FVB Her2 (**g**), FVB C3Tag (**h**), FVB Myc (**i**), and FVB PyMT (**j**). **k** Histogram showing the enrichment score for commonly enriched pathways from MSGE of the C3Tag, Her2, and PyMT models. **l** Histogram showing the fold change of genes common to pathways enriched by MSGE. Unsupervised PCA of individual PT and lung met samples from BL6 (**m**), MOLF (**n**), CAST (**o**), BALB (**p**), SEAGN (**q**), and BL10 (**r**). **s** Volcano plots with significantly up regulated genes in green and down regulated genes in yellow for MSGE in the PyMT-F1 models. **t** Histogram of the number of alternatively expressed genes in lung mets vs. PT for each PyMT-F1 model (FDR < 0.05). **u** Histogram showing the enrichment score for commonly enriched pathways from MSGE of the F1-PyMT models. Histograms showing the fold change of common MSGE enriched in the Focal Adhesion (**v**) and Renin Secretion pathways (**w**).

As MSCNV was not a major contributor to metastasis-specific gene expression (MSGE) within metastatic lesions, we analyzed our RNA-seq data to determine how oncogenic driver or genetic background impacted MSGE. To begin, all samples were visualized together by unsupervised principal component analysis (PCA), which resulted in four distinct clusters differentiated by oncogenic driver (Fig. 5a). When the analysis was limited to individual models within the oncogenic driver cohort there was no significant separation of PT and Lung met samples (Fig. 5b–e). Together, these results indicate that oncogenic driver is a stronger determinant of sample similarity than tissue type or genetic background.

Gene expression analysis comparing Lung met and PTs revealed that the extent to which MSGE differs from the primary tumor is also oncogenic driver-dependent. Using a cut-off of *p* < 0.05 and FDR < 0.1, the PyMT model had significant changes in the expression of 561 genes, C3Tag 113, Her2 64, and Myc only 4 genes (Fig. 5f and Supplementary data 11). While the Her2 and Myc models showed an even distribution of up- and down-regulated genes, the PyMT and C3Tag models favored up-regulation of metastasis-specific genes (Fig. 5g–j). Alternatively, regulated genes in the C3Tag and Her2 models overlapped with those from PyMT, but only a single gene, *Pcdh17*, was commonly altered in metastatic tissue from all four models (Supplementary fig. 8a, b). According to The Genotype-Tissue Expression (GTEx) project, *PCDH17* is highly and specifically expressed in Lung tissue and therefore may simply represent the stroma in which the tumor cells reside (Supplementary fig. 8c). However, this is challenged by the observation that *Pcdh17* is also expressed in PT tissue in a genetic background-dependent manner in the PyMT-F1 cohort (Supplementary fig. 8d). Based on these data, we conclude that the MSGE of these models is largely oncogenic driver-dependent.

Similarly, GO analysis revealed that there were no Lung met-specific gene expression pathways common across all oncogenic driver models (Supplementary fig. 8e and Supplementary data 12). However, it was noted that the Myc model MSGE only enriched a single pathway, the *Wnt Signaling pathway*. Three pathways, *Complement and coagulation cascades, Platelet activation*, and *Malaria*, were significantly enriched and shared by the Her2, C3Tag, and PyMT models (Fig. 5k). Interestingly, while these pathways are related by their involvement with red blood cell biology, the gene lists that define them overlap less than 7% (Supplementary fig. 8f). Upon assessment of the genes within each pathway, we noted that Her2, C3Tag, and PyMT enrich largely independent gene sets except for the cell adhesion molecules *Icam1, Vwf, and Itgb2* (Fig. 5l and Supplementary data 13).

RNA-seq data was next analyzed for metastasis-specific changes in transcript levels to assess model–specific splicing. Upon comparison of Lung mets and PTs from the oncogenic driver cohort, we identified a single transcript significantly altered in metastases compared to PTs and common to all models (Supplementary fig. 9a and Supplementary data 14). *Rnase4* isoform 2, and not isoform 1, was significantly increased in metastatic tissue compared to PT (Supplementary fig. 9b, c). *Rnase4* isoform 1 and 2 share the same promoter and coding exons but have unique 5’ untranslated regions (UTRs)^24,25^. We noted that overall expression of *Rnase4* remained unchanged, which may be explained by a compensatory, although non-significant, decrease of isoform 1 (Supplementary fig. 9b). We asked if Rnase4 might also be alternatively spliced in lung metastases across the PyMT-F1 cohort. A statistically significant increase in levels of isoform 2 was observed in BALB, SEAGN, and BL6, but not any of the remaining strains (Supplementary fig. 9d). Comparative analysis of splicing across the PyMT-F1 cohort revealed no commonly altered transcripts, suggesting that metastasis-specific splicing may be determined in-part by genetic background (Supplementary fig. 9e).

We next asked how genetic background could shape gene expression programs in macro-metastases. Upon visualization of PT and Lung met gene expression by unsupervised PCA for each PyMT-F1 strain, we observed no distinct separation between tissue types for BL6, MOLF, and CAST (Fig. 5m–o), but distinct clustering of samples by tissue in BALB, SEAGN, and BL10 (Fig. 5p–r). The heterozygous PyMT F1s had a higher degree of MSGE compared to homozygous FVB PyMT samples, with the number of altered genes ranging between 814 and 3226 for BL6, BL10, SEAGn, MOLF, and CAST (Fig. 5s, t and Supplementary data 11). Comparison of the MSGE between the F1s using a six-part Venn diagram analysis showed that close genetic background did not always correspond with highest overlap in MSGE (Supplementary fig. 10a). MOLF, BL6, and BALB MSGE were most similar to their corresponding strain pairs, CAST, BL10, and SEAGN, respectively, with about 50% of the MSGE in these strains overlapping with the MSGE of their closely related counterparts. However, SEAGN had the highest overlap in MSGE with CAST, while CAST and BL10 strains had a higher percentage of overlap with one another. Furthermore, we noted that MSGE was also highly individualized between F1 strains, with unique changes in gene expression accounting for 23–58% of MSGE (Supplementary fig. 10a). Only 60 genes were common to MSGE of all strains (1.9–13.6% of total MSGE for each F1 strain), and these were enriched for extracellular matrix, cell adhesion, autophagy factors, and glycoproteins as determined by DAVID analysis (Supplementary data 15)^26,27^.

Further GO analysis was performed using the MSGE for each PyMT-F1 strain (Supplementary data 16). Venn diagram analysis of the enriched pathways revealed *ECM-receptor interaction*, *Gap junction*, *Focal adhesion*, and *Renin Secretion* as four pathways commonly enriched by MSGE across all PyMT-F1s (Fig. 5u). The genes included in the KEGG *ECM-receptor interaction* and *Gap junction* pathways overlap with the *Focal adhesion* pathway by 74% and 30%, respectively, but not one another (Supplementary fig. 10b). As such, when we compared the MSGE of each pathway between strains we identified four commonly altered genes (*Thbs2, Col1a2, Vwf*, and *Egflam*) from the *Focal Adhesion* pathway that were also found within either the *ECM receptor interaction* or *Gap junction* pathways (Fig. 5v and Supplementary data 17). Furthermore, three genes (*Adrb2, Npr1*, and *Acer2*) from the *Renin Secretion* pathway were upregulated in the MSGE of all F1 strains (Fig. 5w). Based on GTex project data, none of these commonly altered genes are Lung-specific, all are expressed in breast tissue, and some are ubiquitously across many tissues (Supplementary fig. 10c). In summary, while the same pathways were enriched by all strains, each F1 appears to modulate gene expression differently to achieve enrichment of these pathways.

### MSCNV and MSGE from both cohorts target common cellular processes

**Fig. 6:**
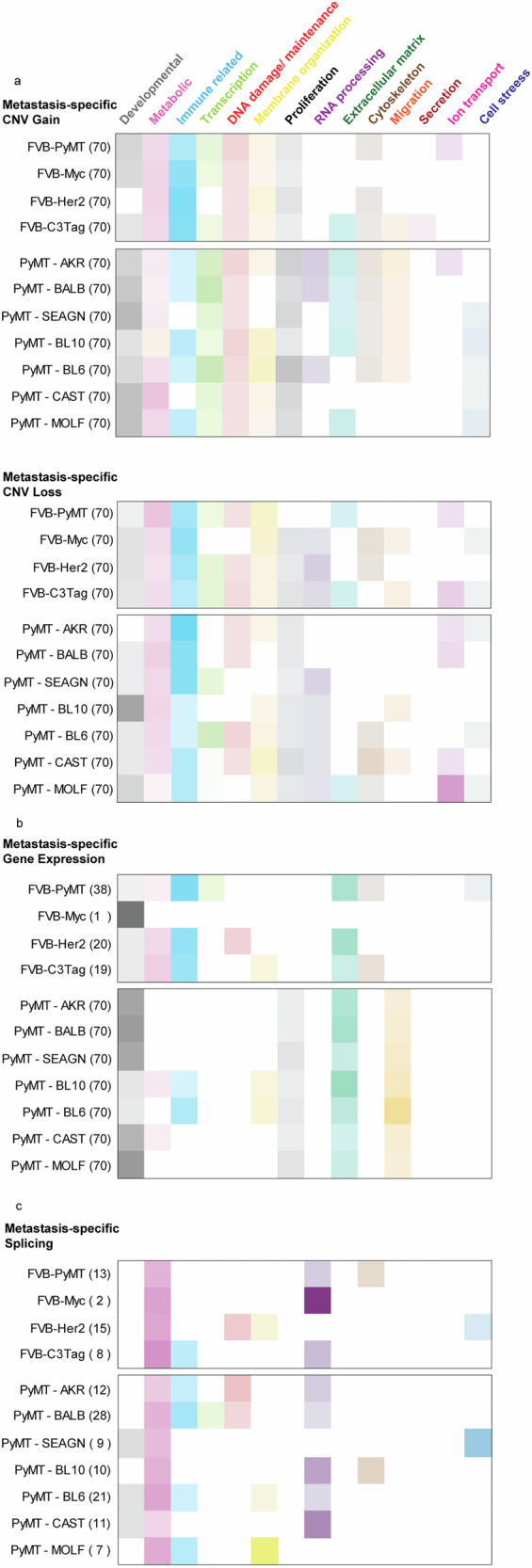
Oncogenic driver and genetic background shape unique MSCNV and MSGE to target common cellular processes. Categorization of the top 70 (or otherwise indicated) significantly enriched gene ontologies from MSCNV (**a**), MSGE (**b**), and lung met-specific alternative splicing (**c**). Uncategorized ontologies were excluded from the heat maps. Color intensity represents the percent of the analyzed pathways that fell within that category.

Analyses conducted in this study sought to identify commonalities between models. However, in many instances, particularly among PyMT-F1s, the models were more unique than similar. Indeed, we noted that the oncogenic driver models had 38–72% of MSCNV enriched pathways that were unique to each model, and the altered genetic background of the PyMT-F1 cohort resulted in no commonly enriched genes or pathways from MSCNV, but rather 60–88% unique pathways in each F1 strain (Fig. 2a and Fig. 3a). To visualize and summarize the differences between models the top 70 GO terms from MSCNV gain and loss were sorted into thirteen categories and the percent of each category within the top pathways presented as heatmaps (Fig. 6a and Supplementary data 7, S9). Immune (cyan) and metabolic (pink) pathways were dominant across all four oncogenic driver models, in both MSCNV gain and loss. However, each model displayed a unique distribution of pathways, and MSCNV gain and loss were also distinct within the same model, which was most pronounced in the C3Tag model (Fig. 6a). MSCNV loss among the PyMT-F1 BALB, AKR, and SEAGN models most closely resembled the distribution observed in the oncogenic driver models, with immune and metabolic pathways dominant, but developmental pathways (gray) were the most significantly enriched across this cohort. From this analysis, we conclude that despite exploiting the same oncogenic driver, genetic background has a stronger influence on the genes enriched within MSCNV (Fig. 6a).

We next extended this analysis to visualize differences in MSGE from RNA-seq data (Fig. 6b). We again observed an enrichment of immune pathways across the oncogenic driver models, except for in the Myc model, which had limited MSGE and only a single enriched pathway (Supplementary fig. 8a and Supplementary data 12, S16). For the PyMT-F1 cohort, we observed more similarity in metastasis-specific gene expression pathways across strains, compared to the wide variety enriched by CNV (Fig. 6a, b). We noted that despite seeing no common developmental pathways, this category made up the majority of MSGE-enriched ontologies among five of the models. In addition, ECM, migration, and proliferative pathways were enriched to varying degrees in the MSGE of each PyMT-F1 strain. Interestingly, the MSGE in the PyMT-F1 cohort represented fewer categories compared to the MSCNV-enriched pathways. Overall, due to the low number of genes and pathways common to MSGE across all PyMT-F1 strains, we conclude that while genetic background can have a profound impact on gene expression within metastatic lesions, these changes may ultimately impact the same cellular processes. This analysis demonstrated that a more diverse genetic background, modeled by the heterozygous PyMT-F1 cohort, shifted MSGE to more developmental and less immune-related ontologies. However, Extracellular matrix (green) genes appear to be commonly modulated by MSGE across all models.

Finally, we assessed pathways enriched by metastasis-specific splicing. In both the oncogenic driver and PyMT-F1 cohorts, we noted that fewer pathways were significantly enriched by alternatively spliced genes compared to MSCNV and MSGE (Fig. 6c and Supplementary data 18). Despite there being no common alternatively spliced transcripts across the eleven models used in this study, we noted that metabolic and RNA processing (purple) ontologies were dominant in all models. However, each model had a unique distribution of pathways significantly enriched by metastasis-specific splicing programs.

## Discussion

Metastasis is a complex phenotype, driven by the interplay of tumor cell intrinsic, microenvironmental, and systemic factors^28^. Classic cytogenetics studies and more recent sequencing-based work has shown that metastases usually arise from a single, or at most a limited number, of subclones within the primary tumor^29^. In addition, clinical and experimental data suggest that only a small fraction of disseminating tumor cells are able to form secondary lesions^30^. These and other data have been interpreted to suggest that tumors evolve over time until a subset of tumor cells acquire all of the necessary attributes to successfully complete the metastatic cascade.

To date, however, in contrast to what has been observed in primary tumors, sequencing studies in clinical samples have not revealed the presence of high frequency, reproducible SNVs associated with metastatic progression^31–33^. In contrast, previous studies from our laboratory, comparing matched primary and metastatic lesions from the highly metastatic MMTV-PyMT mouse model of breast cancer, did identify reproducible metastasis-associated point mutations in *Shc1* and *Kras*. These studies, however, were performed on a much more genetically homogeneous set of animals than is observed in the human population, suggesting that these potential metastasis driver mutations might only represent a small fraction of the patient population. In addition, these observations were made for a single oncogenic driver model, PyMT, rather than the multiple oncogenic initiating events observed for human patients.

To better understand how genomic evolution may contribute to metastatic progression in commonly used GEMMs and to compare them to the expanding clinical data, we have extended our previous work to include two additional models of breast cancer metastasis, the C3(1)TAg and MMTV-Myc models, and expanded on the previously analyzed MMTV-Her2 model. Moreover, for the MMTV-PyMT model, we have expanded the repertoire to include pairs of phylogenetically related strains with significantly different inherited propensity to metastasize to include population diversity into the analysis. The total data set analyzed in this study consists of 197 matched primary and metastatic lesions spanning models of luminal, Her2, and basal-like breast cancer.

Consistent with our previous study, analysis of the expanded data set failed to reveal high frequency SNVs associated with metastasis across all of the models. However, a total of 13 genes were identified that were mutated in 2 or more independent animals, The most frequently observed gene was *Kras*, which had metastasis-associated activating mutations in 5 out of 197 animals. All of the remaining genes with metastasis-associated SNVs were present in only 2 or 3 animals. Reproducible metastasis-associated SNVs were not observed in 84% (166/197) of the animals. This suggests that SNVs do not play a significant role in metastatic progression. A similar result was observed for synchronous lymph node metastasis in human patients^34^, though metastasis-specific SNVs were observed in the metachronous distant metastases. However, since the metachronous lesions were collected after anthracycline treatment, it is unclear whether these SNVs represent metastasis drivers or therapeutic response. A second study by Siegel et al. ^35^ also concluded that the majority of metastasis drivers were copy number alterations, rather than SNVs. Furthermore, Hoadley et al. ^36^ also found few metastasis-specific mutations, which were rarely expressed, and concluded that these SNVs were more likely passengers than drivers, consistent with our results. However, our ability to identify low frequency metastasis-associated SNVs in mouse models, compared to human clinical studies, suggests that there may be either rare SNVs in particular patient populations, or many SNVs that contribute at a low frequency to metastatic progression, which are more readily apparent through sequencing of multiple genetically identical independent sample pairs. Deeper sequencing and significantly increasing the number of samples per group may enable the identification of additional low frequency metastasis drivers, particularly those that might be selectively lost at the secondary site due to clonal selection. The mouse data generated here, however, is consistent with current clinical data suggesting that there is a lack of high frequency activating mutations for precision medicine for anti-metastatic therapies that would be of benefit for significant fractions of breast cancer patients.

We subsequently performed CNV analysis on the data to determine whether there were common CNVs that might contribute to metastatic progression. Analysis revealed that oncogenic drivers on the FVB homozygous genetic background had similar degrees of CNV in both primary and secondary lesions, though there were differences in the size of the CNVs between models. Further, in both PTs and lung metastases we observed some strains with high numbers of small events and others with small numbers of larger events, accounting for the similar degree of copy number change overall. Notably, the size of copy number events in this study are within the range observed across cancer types by Steele et al. in a recently published analysis of copy number signatures from clinical data^37^. The major determinant of CNV variation, however, was genetic background. This was most apparent in the comparison of the phylogenetically related BALB and SEAGn strains. The SEAGn strain was generated by a cross between BALB and PJ mice, followed by 20 generations of brother-sister mating^38^. This congenic strain genome is 50% BALB yet shows significantly less genomic stability compared to the BALB primary tumor. Together this data suggests that genetic background may direct the mechanisms leading to copy number events.

Further analysis focused on metastasis-associated events revealed common CNVs, associated genes, and gene ontologies across the oncogene driver models on the FVB homozygous background. However, these common metastasis-associated features are lost when the genetic background was varied in the MMTV-PyMT cohort. This suggests that while the analysis of the four homozygous FVB oncogenic models may be valuable for identification of some common metastatic mechanisms, they are unlikely to represent the majority of the human patient population. Moreover, consistent with our previous observations of inherited metastatic susceptibility, this suggests that an individual’s personal ancestry is a major factor in metastatic genome evolution. In addition, these data suggest that there are likely multiple paths that tumor cells utilize to achieve metastatic competency, which indicates that multiple strategies to prevent or treat established lesions may be required across the human population.

Similar to the CNV analysis, transcriptional analysis demonstrated that the most significant determinants of gene expression were oncogenic driver and genetic background. Surprisingly, CNV status did not show strong correlation with metastasis-specific gene expression when analyzing macroscopic metastatic lesions. One possible explanation for this is that alterations in gene expression due to CNV are important during the earliest stages of dissemination, extravasation and adaptation to the secondary site. Once resident in the secondary site epigenetic regulation mediated by microenvironmental influences of the pre-metastatic niche and metastatic tumor bed may reprogram and dominate the transcriptional program of the macroscopic metastatic tumors. Additional investigations into the epigenetic state of the matched primary and metastatic tumors in these models, however, will be necessary to address this possibility.

**Fig. 7:**
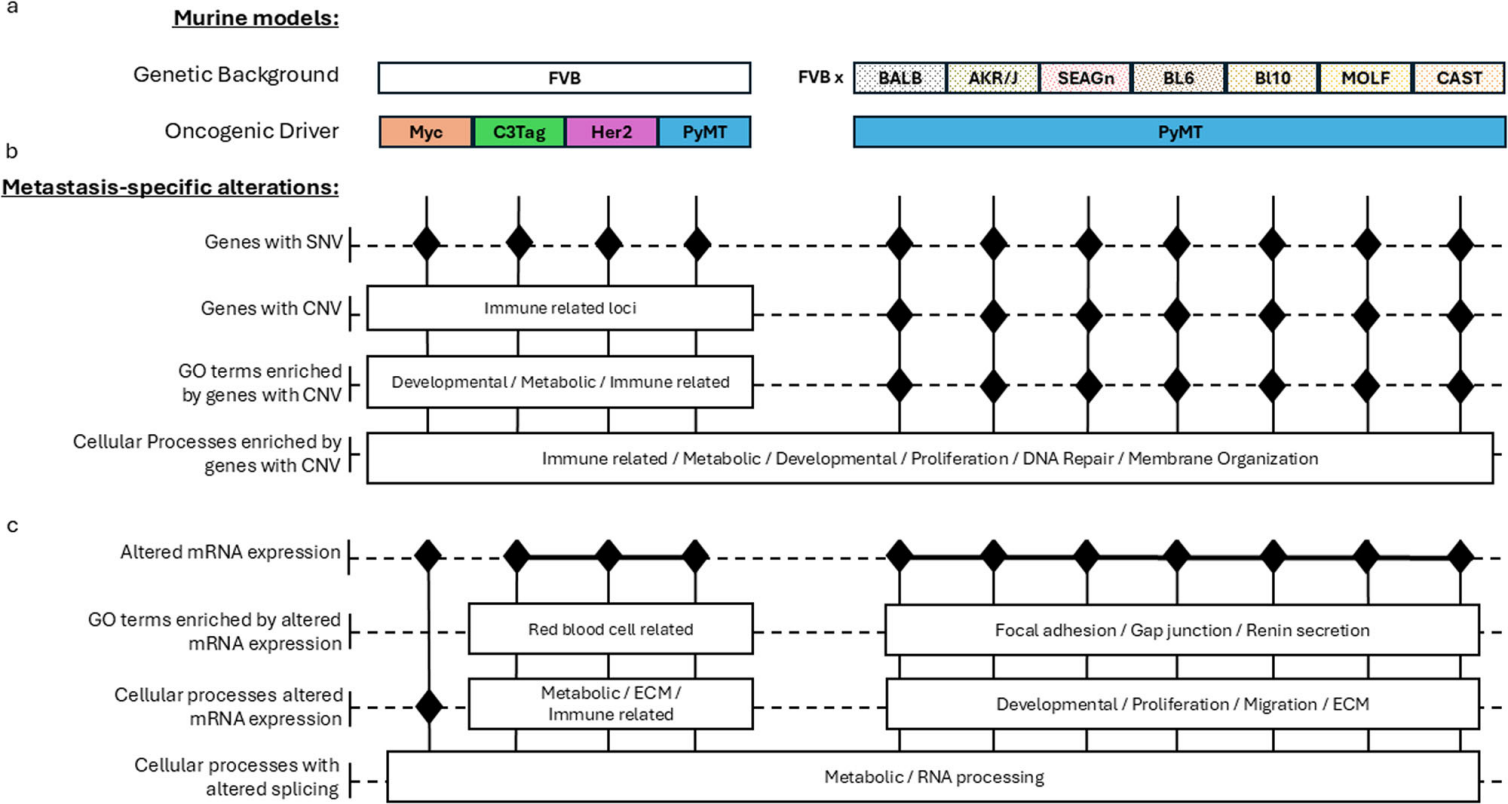
Host genetic background shapes metastatic SNVs, CNVs, and gene expression more than oncogenic driver. Schema summarizing **a** murine model genetic background and oncogenic driver and the unique (black diamonds) or common (white boxes and solid lines) metastasis specific **b** genomic alterations and **c** gene expression.

Examination of the data at a broader level provides cause for optimism. Although individual Gene Ontology terms from CNV and transcriptome analysis were not shared across the models and genotypes, grouping individual ontologies into broader categories demonstrates potential commonalities that might be targeted. When considered as broad categories, developmental pathways, ECM, RNA biology, and metabolism appear to be common metastatic categories across all of the animals, although different Gene Ontologies are affected in each of the cohorts (Fig. 7). Each of these broad categories have already been previously associated with metastatic disease. Importantly, a recent sequencing study also identified metabolism and RNA biology ontologies distinguishing matched primary and metastatic breast cancer samples^35^, providing greater confidence that the results reported here are translationally relevant. The data presented here suggests that to generate therapies that have broad applicability across patient populations more in-depth analysis of these categories may be necessary. In particular, since the models used here appear to rely on different pathways within each of the broad ontology categories, targeting any single pathway is unlikely to be efficacious in a large percentage of the patient population. Further investigations into pathway intersections within the broad metastasis categories, however, might identify critical convergence points that might be targeted in either single or combination therapies to suppress metastasis establishment or metastatic lesion viability.

This study has focused on the characterization of the genomic evolution of commonly used mouse models of breast cancer progression and metastasis. A major strength of this study is the ability to generate multiple genetically identical animals for each model and genotype, enabling more accurate dissection of the role of oncogenic driver and genetic background on tumor evolution and transcriptional output. Limitations of this study include potential biological differences between the two species. In addition, many of the features observed in these models recapitulate events observed in human cancer progression. For example, human tumors also experience significant copy number alterations, with up to 25% of the genome of all human tumors acquiring somatic amplifications of deletions. Moreover, like the mouse tumors examined here, human tumors tend to accumulate a higher number of copy-number alterations than single nucleotide mutations^39^. Furthermore, human circulating tumor cells often have copy-number alterations that more closely resemble metastatic lesions rather than the primary tumors^40^, suggesting that copy-number alteration provides a selective advantage for metastatic progression. Increased copy-number alteration has also been associated with increased probability of metastasis and mortality in prostate cancer^41^, consistent with the data presented here.

Limitations of this study, however, include potential differences between the two species. Mouse and human immune and metabolic systems are known to have differences, which may potentially contribute to differences in the important tumor-stromal cell interaction during metastatic dissemination and colonization. In addition, the mechanism of copy-number alteration may differ between mouse and human. Whole genome duplication is a frequent event in human metastatic tumors^23^. However, analysis of the primary and metastatic lesions in the mouse models suggests that these tumors had not experienced whole genome duplication. In addition, although the use of multiple inbred strains has introduced genetic diversity for the MMTV-PyMT model, the remaining three models studied here were limited to the FVB/N homozygous background, which precluded the ability to investigate the role of genetic diversity in these models. In addition, the metastases examined here were limited to pulmonary lesions and for most animals limited to a single metastatic lesion per animal. This precluded investigations into the potential tissue-specific differences as well as performing phylogenetic tracing analysis.

Despite these limitations, here we have demonstrated that (1) Genetic background plays a role in primary tumor stability and the size of copy number event regions, (2) Genetic background is a strong determinant of which genes are specifically enriched in CNV, (3) Oncogenic driver and genetic background shapes gene expression in metastatic tumors, and (4) Metastasis-specific ontology clusters, rather than individual Gene Ontologies, identify common biological programs in metastatic disease that might be considered for clinical intervention (Fig. 7). An increased understanding of the complex interplay between these factors will provide guidance for the improved use and interpretation of these models in relation to human patient populations for the investigation of progressive metastatic disease.

## Methods

### Ethics statement

We have complied with all relevant ethical regulations for animal use. The research described in this study was performed under the Animal Study Protocol LPG-002, approved by the National Cancer Institute (NCI) Animal Use and Care Committee. Animal euthanasia was performed by cervical dislocation after anesthesia by Avertin.

### Genetically engineered mouse models and tissue isolation

FVB/N-Tg(MMTV-PyVT)634Mul/J (PyMT), FVB/N-Tg(MMTVneu)202Mul/J (Her2) male mice were obtained from The Jackson Laboratory. FVB/N-Tg(C3(1)-TAg) (C3-TAG) and FVB/N-Tg(MMTV-Myc) (MYC) male mice were a generous gift from Dr. Jeffrey Green (NCI, Bethesda, MD). Male PyMT mice were crossed with female wild-type FVB/NJ, MOLF/EiJ, CAST/EiJ, C57BL/6J, C57BL10/J, BALB/cJ, Sea/GnJ, and AKR/J mice, which were also obtained from The Jackson Laboratory. Male Her2, C3-TAG, and MYC mice were crossed with female wild-type FVB/NJ mice. All female F1 progeny were genotyped by the Laboratory of Cancer Biology and Genetics genotype core for the PyMT, Her2, Myc, or C3-TAG gene. Transgene-positive female animals were housed in standard isolator cages and provided food and water *ad libitum*. PyMT-positive female animals were aged to permit tumor initiation and growth until animals reached humane endpoints or were expected to reach humane endpoints by the next daily monitoring. For the MMTV-Myc, MMTV-Her2 and C3(1)-TAg models, tumors from animals with single lesions were surgically resected at or before tumors reached 1 cm in any direction. Animals were then aged an additional 60 days or until they approached humane endpoints, whichever came first. All animals were housed in the same room in the building 37 vivarium. Mice were euthanized using intraperitoneal Avertin to anesthetize followed by cervical dislocation. All primary tumors generated by one animal were isolated, weighed, randomly sampled, and combined into a single cryovial. Metastatic nodules and normal (tail) tissue were also isolated immediately following euthanasia, placed in cryovials, and snap frozen in liquid nitrogen. Tissue samples were then stored at −80 °C.

Humane endpoints included hunched posture, rough hair coat; dehydration (reduced skin turgor, sunken eyes); rapid or labored breathing, dyspnea, coughing; reduced/impaired mobility affecting the ability to obtain food or water; pallor or cyanosis (evident in the general appearance of the feet, muzzle or ears which may turn white or blue); hemorrhage or bleeding from any orifice; diarrhea, constipation or markedly reduced food intake; signs of neurological impairment for example seizures, paralysis, circling, head tilt; impaired ability to urinate or defecate; visible jaundice (yellow discoloration to ears, muzzle, or feet); loss of >15% normal body weight. Additional humane endpoints included tumors interfering with normal movement that impaired their ability to perform bodily functions, significantly impaired gait, or impaired their ability to obtain food or water; tumors interfering with normal bodily functions such as urination, defecation, breathing, chewing, or swallowing; or dyspnea at cage side observation.

### gDNA and RNA isolation

The combined primary tumor tissue from one mouse was ground on dry ice and small fragments were taken for nucleotide extraction isolation. Whole metastases were used for gDNA or RNA isolation.

For gDNA isolation, tissue was lysed using Tail Lysis Buffer (100 mM Tris-HCl pH 8.0, 5 mM EDTA, 0.2% SDS, 200 mM NaCl, 0.4 mg/ml proteinase K) at 55 °C overnight. Samples were then placed in a shaking (1400 rpm) heat block for 1 h at 55 °C. RNaseA (Thermo Fisher Scientific) was added (2 mg/ml final) and lysates were incubated on the bench for 2 min. gDNA was then isolated using the ZR-Duet DNA/RNA MiniPrep kit (Zymo Research).

For RNA isolation, the tissue was mechanically dissociated using a tissue grinder while submerged in 1 ml of TriPure (Roche). 200 μl of chloroform (Sigma-Aldrich) was then added and the soluble fraction was isolated by centrifugation at 12,000 rpm for 15 min at 4 °C. RNA was then precipitated with the addition of 500 μl isopropanol and incubation of the sample at −20 °C for 2 h. Pure RNA was then extracted using the RNA: DNA mini-prep kit (Zymogen) and samples were eluted in 100 μl (PT) or 50 μl (metastases) of DEPC water (Quality Biological). RNA was isolated from cell lines using TriPure as described above, but following isopropanol precipitation, RNA was washed with 75% ethanol (Sigma-Aldrich) followed by 95% ethanol before being resuspended in 100 μl of DEPC water.

All samples that passed initial quality control were used for sequencing analysis. No samples were excluded for any reason other than low yield or poor quality. No randomization was performed.

### Sequencing and analysis

All analyses were carried out on the NIH Biowulf2 high performance computing environment. All analyses were performed using software default parameters if not otherwise specified.

#### Exome sequencing and data analysis

Exome sequencing was performed by the NCI Center for Cancer Research (CCR) Genomics Core and the NCI Illumina Sequencing Core. Exome libraries were prepared by the Genomics Lab using Agilent SureSelectXT Mouse All Exon target enrichment kit. Libraries were barcoded and pooled before sequencing on an Illumina HiSeq3000 or HiSeq4000 to an average depth of 40×. Samples were trimmed of adapters using Trimmomatic software. Bam files were uploaded into Nexus Copy Number 10.0 (BioDiscovery) and processed using SNP-FASST2 Segmentation. The trimmed reads from tumor gDNA were aligned to normal tail gDNA sequence from each model, and then to the mouse NCBI build 38 (mm10) reference genome.

#### RNA sequencing and data analysis

RNA quality was tested using the Agilent 2200 TapeStation electrophoresis system, and samples with an RNA integrity number (RIN) score >7 were sent to the Sequencing Facility at Frederick National Laboratory. Preparation of mRNA libraries and mRNA sequencing was performed by the Sequencing Facility using the HiSeq2500 instrument with Illumina TruSeq v4 chemistry. RNA-seq data were mapped to mm10 and analyzed using Partek Flow software.

#### Single nucleotide variant analysis

This analysis was performed as described in our previously published work^6^.

#### Copy number analysis

Following initial data processing, the *Total copy number events* and *% of the genome changed* by copy number for each sample were downloaded from the Data Set tab. Aggregate data from PTs and lung mets was attained by selecting only the sample from the desired tissue type within the Data set tab, and then selecting *View* and then *Aggregate*. Aggregate regions of CNV were defined as having a *p* value cutoff below 0.05 and an aggregate percent frequency of 35% and above. Region length, event type, number of genes, and gene names were exported as .txt from aggregate analysis. Average region length, total number of events and total number of genes were calculated using Excel (Microsoft).

For lung met-specific CNV analysis, the *Classic* comparison tool was utilized in Nexus. In the Data Set tab, all samples from one mouse model were selected before navigation to the *Comparisons* tab. From here, a comparison was added using *Tissue* as the distinguishing factor, and *Average* was selected as the comparison baseline. Upon selection of the comparison and then *View*, lung met specific *e*vent type, region length, *p* value, number of genes, and gene names were exported as .txt files. Average region length, total number of events, and total number of genes were calculated using Excel. By highlighting specific CNV events within the comparison V*iew* tab, a pathway enrichment analysis was performed for the genes found within regions of CN Gain or CN Loss. A list of enriched *Biological Processes* was exported as a .txt file including *p* values. The percent CN gain, loss, or allelic imbalance in the primary tumors and lung mets for each gene within each pathway was obtained by selecting the individual biological process name. Calculation of % Lung met gain or loss − % PT gain or loss was performed in Excel and heat maps were created using the Excel conditional formatting tool.

#### ABSOLUTE whole genome duplication analysis

Reference files were trimmed using the Trim galore^42^, before being aligned to the mm10 genome using the Bwa-mem2 package^42,43^. We then used Gatk to sort, deduplicate, and recalibrate the resulting files^44^. Using Gatk’s Mutect2 somatic variant caller, SNPs were called for all tumor-normal pairs. The R packages copywriter and DNAcopy were used to detect Copy Number Aberrations within tumor samples and format the data for integration with our SNP data^45,46^. We used the R package BubbleTree to integrate CNA and SNP information to analyze tumor purity and ploidy^47^.

#### RNA sequencing and data analysis

RNA quality was tested using the Agilent 2200 TapeStation electrophoresis system, and samples with an RNA integrity number (RIN) score >7 were sent to the Sequencing Facility at Frederick National Laboratory. Preparation of mRNA libraries and mRNA sequencing was performed by the Sequencing Facility using the HiSeq2500 instrument with Illumina TruSeq v4 chemistry.

RNA-seq data was analyzed using Partek Flow software (Kanehisa Laboratories). RNA-seq reads were uploaded into Partek Flow, aligned with the mouse mm9 genome assembly, gene counts were then determined, and then normalized. Principal Component Analysis was performed using unsupervised principal component analysis for either all samples, or samples filtered by mouse model. Differentially expressed gene lists were generated Gene Specific Analysis (GSA) for FVB-Driver models and Dseq2 for PyMT-F1 models. Differential gene expression was defined as a fold change with a false discovery rate less than 0.05. The Differential expression of genes within regions of CNV was defined as fold change with FDR less than 0.1. Volcano plots for Lung met v PT gene expression were created using unfiltered GSA results for all models. Gene ontology analysis was performed using differentially expressed gene lists and the Partek Flow Gene Set Enrichment tool. Pathways enriched with a *p* value less than 0.05 were considered significant. Gene ontology categories were assigned manually to the top 70 pathways or if less than 70 pathways were significantly enriched then all pathways with *p* < 0.05 were included. Heatmaps were created using the Excel conditional formatting tool, where color intensities were determined by the percent of the enriched terms found within each category out of the total terms categorized.

Differential splicing analysis was performed using Partek flow. Following alignment to mm9 genome assembly, transcript counts were normalized and then each model was analyzed using the Partek tool to detect all splicing and differential splicing in Lung mets v PT. Alternative splicing was defined as both transcript differential expression with *p* value less than 0.05 and an alt-splicing *p* value less than 0.05. Using these *p* value cuts-offs, alternatively spliced gene lists were analyzed by Partek Gene Set Enrichment to identify pathways enriched by lung met alternatively spliced genes. Significantly enriched pathways were defined as those with *p* values less than 0.05.

### Statistics and reproducibility

Statistical analysis was performed using GraphPad Prizm software, using *t* tests or ANOVA analysis with correction for multiple testing, as required. Sample numbers can be found in Supplementary Table 1.

### Reporting summary

Further information on research design is available in the Nature Portfolio Reporting Summary linked to this article.

## Supplementary information

Supplementary Figs.

Supplementary data 1–18

Description of Additional Supplementary Files

Reporting Summary

## Data Availability

The sequencing data described in this study are available from the Sequence Read Archive (PRJNA1090863) or the Gene Expression Omnibus (GSE142387).
