# Supplementary material for: Genetic background and oncogenic driver determines the genomic evolution and transcriptomics of mammary tumor metastasis
Source: Commun Biol. 2025 Aug 14;8:1224. doi: 10.1038/s42003-025-08624-5 (PMC12354892; doi:10.1038/s42003-025-08624-5)
Supplement: Supplementary file 2 — Supplementary data 1–18 [file 42003_2025_8624_MOESM2_ESM.zip › Supplementary Data/Supplementary Table Legends.docx]

**Supplementary Tables**

Supplementary table 1 - Sample numbers and attributes

Supplementary table 2 - Total number of CN aberrations and % genome with CNV for all models.

Supplementary table 3 - Type, size, and loci for aggregate CNVs in FVB-driver model PTs (Sheet 1) and Lung mets (Sheet 2), and for PyMT-F1 cohort PTs (Sheet 3) and Lung mets (Sheet 4).

Supplementary table 4 - Type and length of MSCNV for FVB-driver (Sheet 1) and PyMT-F1 (Sheet 2) cohorts.

Supplementary table 5 - MSCNV gene lists for FVB-driver (Sheet 1) and PyMT-F1(Sheet 2) cohorts.

Supplementary table 6 - DAVID functional clustering annotation analysis using 1676 common MSCNV gene list from FVB driver models.

Supplementary table 7 - GO analysis for genes within MSCNV for each FVB driver model. PyMT (Sheet1), Her2 (Sheet 2), C3Tag (Sheet 3), Myc (Sheet4). Overlapping GO terms with assigned category and -Log10(p values) (Sheet 5).

Supplementary table 8 - FVB Driver Model Heat Maps separated by pathway category. Immune Pathways (Sheet1), Metabolic Pathways (Sheet2), Developmental pathways (Sheet 3), and Other Pathways (Sheet4). Blue indicates genomic gain and red indicates genomic loss in metastatic tissue vs PT.

Supplementary table 9 - GO analysis for MSCNV-enriched genes for each F1 of the PyMT-F1 cohort. Separated by sheets 1-7.

Supplementary table 10 - Gene expression overlaps with MSCNV for each model.

Supplementary table 11 - MSGE for each individual model separated by sheets 1-11.

Supplementary table 12 - Oncogenic Driver cohort GO enrichment in MSGE for each driver model separated by sheets 1-4.

Supplementary table 13 - MSGE of FVB-PyMT, Her2, and C3Tag common pathways as heatmaps. Indicating upregulation (green), down regulation (yellow), and significance (red text) for each gene within each model.

Supplementary table 14 - Metastasis-specific splicing for each individual model separated by sheets 1-11.

Supplementary table 15 - DAVID functional clustering annotation analysis using PyMT-F1 MSGE overlap.

Supplementary table 16 - PyMT-F1 cohort GO enrichment in MSGE for each F1 model separated by sheets 1-6

Supplementary table 17 - MSGE of F1-PyMT common pathways as heatmaps. Indicating upregulation (green), down regulation (yellow), and significance (red text) for each gene within each model.

Supplementary table 18 - GO analysis of MS splicing for each model separated by sheets 1-11.

Supplementary table 19 – Statistical summary of data analyses in Figure 1 and Figure S3.
