# Supplementary material for: Genetic background and oncogenic driver determines the genomic evolution and transcriptomics of mammary tumor metastasis
Source: Commun Biol. 2025 Aug 14;8:1224. doi: 10.1038/s42003-025-08624-5 (PMC12354892; doi:10.1038/s42003-025-08624-5)
Supplement: Supplementary file 3 — Description of Additional Supplementary Files [file 42003_2025_8624_MOESM3_ESM.docx]

Description of Additional Supplementary Files

**File name:** Supplementary Data

**Description:** Combined figure panel data tabs.
